# Cold acclimation by the CBF–*COR* pathway in a changing climate: Lessons from *Arabidopsis thaliana*

**DOI:** 10.1007/s00299-019-02376-3

**Published:** 2019-01-16

**Authors:** Yukun Liu, Peiyu Dang, Lixia Liu, Chengzhong He

**Affiliations:** 1 Key Laboratory of Forest Resources Conservation and Utilization in the Southwest Mountains of China (Southwest Forestry University), Ministry of Education, College of Forestry, Southwest Forestry University, 300 Bailong Si, Kunming, 650224 Yunnan People’s Republic of China; 2 School of Ecology and Landscape Architecture, Dezhou University, 566 West University Road, Dezhou, 253023 Shandong People’s Republic of China; 3 Key Laboratory for Forest Genetic and Tree Improvement and Propagation in Universities of Yunnan Province, School of Life Sciences, Southwest Forestry University, 300 Bailong Si, Kunming, 650224 Yunnan People’s Republic of China

**Keywords:** CBFs, CAMTAs, Protein kinase, Cold-responsive gene, Abiotic stress, Local adaptation

## Abstract

Cold acclimation is a process used by most temperate plants to cope with freezing stress. In this process, the expression of cold-responsive (*COR*) genes is activated and the genes undergo physiological changes in response to the exposure to low, non-freezing temperatures and other environmental signals. The C-repeat-binding factors (CBFs) have been demonstrated to regulate the expression of many *COR* genes. Recent studies have elucidated the molecular mechanisms of how plants transmit cold signals from the plasma membrane to the CBFs and the results have indicated that *COR* genes are also regulated through CBF-independent pathways. Climate change is expected to have a major impact on cold acclimation and freezing tolerance of plants. However, how climate change affects plant cold acclimation at the molecular level remains unclear. This mini-review focuses on recent advances in cold acclimation in *Arabidopsis thaliana* and discusses how signaling can be potentially impacted by climate change. Understanding how plants acquire cold acclimation is valuable for the improvement of the freezing tolerance in plants and for predicting the effects of climate change on plant distribution and agricultural yield.

## Introduction

Cold temperature (chilling or freezing) is a recurring phenomenon that limits the geographical distribution and agricultural yield of plants. Cold exerts adverse effects on most plant species and causes cold stress. Over the course of their evolutionary history, plants developed different strategies to adapt to cold stress (Korner 2016). Most freezing-tolerant plants acquire this ability via cold acclimation, through exposure to low temperatures that remain above freezing (Thomashow 1999). Experimental studies showed that acquisition of cold acclimation requires the orchestration of transcriptional, biochemical, and physiological changes. During cold acclimation, C-repeat binding factors (CBFs) activate cold-responsive (*COR*) genes and subsequent accumulation of cryoprotectants, which results in the acquisition of freezing tolerance (Thomashow 1999). Under natural conditions, cold acclimation is a plant response that ensures seasonal survive of low winter temperatures. Cold acclimation, often associated with decreasing photoperiod, initiates the cessation of tree growth in winter and freezing tolerance (Maurya and Bhalerao 2017). Climate change causes rapid temperature changes combined with increasing atmospheric CO_2_ concentrations (Shepherd 2016), which impacts plant cold acclimation and freezing tolerance. This review focuses on how the cold signal is sensed and transduced into the nucleus and the potential impact of climate change on plant cold acclimation is discussed.

## Regulation of *COR* genes by CBF-dependent and CBF-independent pathways

**Fig. 1:**
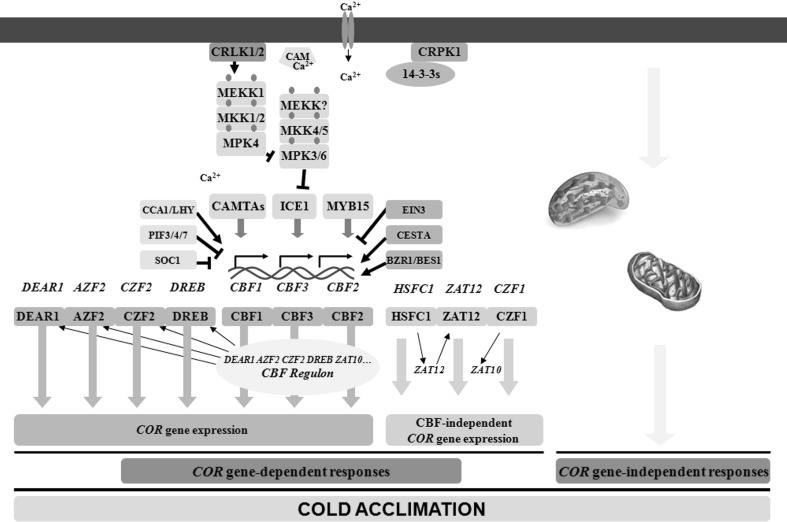
Proposed model of signal-induced cold acclimation. Plants acquire cold acclimation through *COR* gene-dependent and *COR* gene-independent responses. Acquisition of *COR* gene expression is categorized into CBF-dependent and CBF-independent pathways. CBFs have been identified as master transcription factors that regulate the expression of many *COR* genes, including *DEAR1, DREB, ZF, CZF2, ZAT10*, and *AZF2* whose proteins further regulate many *COR* genes. Expression of *HSFC1, ZAT12*, and *CZF1* is also rapidly induced by cold stress and is involved in the regulation of *COR* gene expression. Functional redundancy and likely inter-regulation exist among CBF transcription factors. In turn, *CBF* expression is controlled by other transcription factors, e.g., ICE1, SOC1, MYB15, and CAMTAs. Upstream events include cold-induced calcium influx, enhanced membrane rigidity, activation of protein kinases, and balanced control between protein activation and degradation. These post-translational mechanisms guarantee rapid activation of the CBF transcriptional pathway during cold acclimation and inactivation of the pathway once *COR* gene expression has been initiated. *AZF2* Arabidopsis zinc-finger protein 2, *BES1* brassinosteroid-insensitive 1-EMS-suppressor 1, *BZR1* brassinazole-resistant 1, *CAM* Ca^2+^/calmodulin, *CAMTAs* calmodulin-binding transcription activators, *CBF* C-repeat binding factor, *CCA1* circadian clock-associated 1, *CESTA* a bHLH transcription factor, *COR* cold responsive; CRLK1/2, calcium/calmodulin-regulated receptor-like kinases 1 and 2, *CRPK1* cold-responsive protein kinase 1, *CZF* cold-induced zinc-finger protein 2, *DEAR1* DREB and EAR motif protein 1, *DREB* dehydration-responsive element-binding protein, *EIN3* ethylene-insensitive 3, *HSFC1* heat-shock factor C 1, *ICE1* inducer of CBF expression 1, *LHY* late elongated hypocotyl, *MEKK* mitogen-activated protein kinase kinase kinase, *MKK* mitogen-activated protein kinase kinase, *MPK* mitogen-activated protein kinase, *MYB15* MYB transcription factor 15, *PIF3/4/7* phytochrome-interacting factor 3, 4 and 7, *SOC1* suppressor of constans overexpression 1, *ZAT* zinc finger of *Arabidopsis*

Overall, cold acclimation is a result of both *COR* gene-dependent and *COR* gene-independent responses. Expression of *COR* genes can be regulated through CBF-dependent and CBF-independent pathways (Fig. 1). *COR* genes are rapidly induced (ranging from minutes to several hours) by low temperature during cold acclimation (Thomashow 1999). Many products of the *COR* genes have been suggested to function in the acquisition of cold acclimation and subsequent freezing tolerance. These products include enzymes to biosynthesize osmo-protectants, late embryogenesis abundant proteins, transcription factors, protein kinases, proteins associated with lipid metabolism, proteins for hormone responses, cell wall modifiers, and chloroplast proteins. A 24-h treatment at 4 °C induces about 4000 *COR* genes in *Arabidopsis thaliana* (Zhao et al. 2016). CBF1, CBF2, and CBF3 (also known as DREB1b, DREB1c, and DREB1a, respectively; Kidokoro et al. 2017) regulate about 10% of all *COR* genes (Park et al. 2015). It has been shown that the genes induced or repressed by each CBF are very similar, suggesting that the three CBF proteins are partly redundant in regulating the *COR* genes (Park et al. 2015; Jia et al. 2016; Zhao et al. 2016; Shi et al. 2017). However, distinct functions of CBFs have also been reported, indicated by the differential expression patterns of the *CBF* genes during cold acclimation (Shi et al. 2017).

The CBF proteins directly regulate *COR* genes by the CCGAC *cis*-acting element known as the C-repeat (CRT)/dehydration-responsive element (Thomashow 1999). However, not all CBF-regulated *COR* genes are directly regulated by CBF proteins. Analysis of the promoters of the CBF-activated *COR* genes in *A. thaliana* showed that about 38% have no CRT in the 1000 bp upstream of the ATG start codon (Zhao et al. 2016). Furthermore, expression of few CBF-regulated *COR* genes with or without CRT is repressed, indicating that more transcription factors are involved in the regulation of CBF-regulated *COR* genes. In addition to *CBFs*, expression of the other 27 first-wave (rapidly induced in parallel with *CBFs*) transcription factors are also induced during cold acclimation in *A. thaliana* (Vogel et al. 2005; Park et al. 2015; Zhao et al. 2016). Six of them (*DEAR1, DREB, ZF, CZF2, ZAT10*, and *AZF2)* are significantly repressed in the *cbf1*/*2*/*3* triple mutant, indicating that the cold-induced expression of these genes is CBF dependent (Zhao et al. 2016). The functions of *ZF* and *ZAT10* were tested via transgenic expression. Overexpression of each induces the expression of *COR* genes even without cold treatment, suggesting that their transcriptional activities are involved in regulating *COR* genes (Park et al. 2015; Zhao et al. 2016).

The CBF-independent pathway is involved in the regulation of *COR* genes, as not all *COR* genes are affected by *CBF* genes (i.e., in single, double, or triple *cbf* mutants of *A. thaliana*). Among the 27 first-wave transcription factors, HSFC1, ZAT12, and CZF1 regulate the expression of *COR* genes, but their expression is not affected in *cbf* triple mutants (Park et al. 2015; Jia et al. 2016; Zhao et al. 2016; Shi et al. 2017). Since only 11 of the 27 first-wave transcription factors have been tested, it is possible that additional CBF-independent transcription factors are involved in the regulation of *COR* gene expression.

The *COR* gene expression is complex because two or more first-wave transcription factors share common downstream genes (Park et al. 2015; Zhao et al. 2016). The regulatory network of *COR* genes is highly interconnected and involves both extensive crosstalk and co-regulation. The regulatory network extends to genes encoding the transcription factors themselves, e.g., CBF2 regulates the expression of *ZF*, HSFC1 regulates the expression of *ZAT12*, and CZF1 regulates the expression of *ZAT10* (Park et al. 2015; Zhao et al. 2016). Therefore, it seems that cold acclimation is orchestrated by several master proteins and facilitated by other transcription factors, where a coordinated signaling and regulatory network leads to rapid changes of transcriptome.

## Expression and regulation of *CBF* genes

In cold acclimation, the CBF-dependent pathway has been recognized as key to regulate the expression of many *COR* genes. In turn, *CBFs* can also be rapidly induced by low temperature during cold acclimation (Thomashow 1999). *CBFs* have been identified as a gene family in plants and cold induces different expression patterns of different *CBF* members with regard to specific expression and kinetics (Tondelli et al. 2011). In addition, the expression of *CBFs* is regulated by light quality, the circadian clock, and photoperiod under normal (e.g., 22 °C) temperatures. Cold-induced *CBF* expression can be affected by light quality, the circadian clock, and photoperiod. The phytochrome-interacting factor 3/4/7 (PIF3/4/7) directly binds to *CBF* promoters in *A. thaliana* and negatively regulate *CBF* expression, whereas circadian clock-associated 1 (CCA1) and late elongated hypocotyl (LHY) directly bind to *CBF* promoters and positively regulate *CBF* expression (Kidokoro et al. 2009; Dong et al. 2011; Lee and Thomashow 2012; Jiang et al. 2017). During cold acclimation, pseudo-response regulator 5/7/9 (PRR5/7/9) is implicated in repressing *CBF* expression by affecting the expression of *CCA1* and *LHY* (Nakamichi et al. 2009). The decrease in the red to far-red (R/FR) ratio increases *CBF* expression (Franklin and Whitelam 2007). COR27 and COR28 are nighttime repressors (Wang et al. 2017). Blue light-repressed *COR27* and *COR28* have been shown to negatively regulate *CBF* expression through crosstalk with CCA1 function and with *PRR5* expression via unknown mechanisms (Li et al. 2016). Recent studies reported that circadian regulation of *CBF* expression includes plastid signals. LONG HYPOCOTYL 5 (HY5) and PRR5 repress basal expression of *CBFs*. Specifically, HY5 represses the expression of *CBF3*. The molecular chaperone HSP90 directly controls the F-box protein ZEITLUPE (ZTL) to negatively regulate HY5 and PRR5. In turn, the heat-shock protein 90 (HSP90)–ZTL complex is negatively regulated by a plastid signal triggered by tetrapyrrole accumulation, providing a signaling cascade that regulates nuclear expression of *CBF* genes using tetrapyrrole accumulation (Noren et al. 2016).

During cold acclimation, several transcription factors have been identified to regulate the expression of *CBFs* by binding to their promoters. In *A. thaliana*, inducer of CBF expression 1 (ICE1) is an MYC-like basic helix–loop–helix transcription factor that binds to the MYC *cis*-acting elements in the *CBF* promoter (Chinnusamy et al. 2003; Ding et al. 2015; Kim et al. 2015). The function of ICE1 depends on its post-translational modification but not gene expression (Chinnusamy et al. 2003; Miura et al. 2007, 2011; Ding et al. 2015, 2018). Recent reports showed that ICE1 is regulated by mitogen-activated protein kinase (MPK) signaling cascades that typically comprise three protein kinases: MEKK, MKK, and MPK, which act in series (i.e., MEKK–MKK–MPK; Liu 2012; Liu and He 2017). The MKK4/5–MPK3/6 pathway promotes degradation of ICE1 and repression of *CBF* genes (Li et al. 2017; Zhao et al. 2017). During cold acclimation, the cold signal causes calcium influx that activates calcium/calmodulin-regulated receptor-like kinases 1 and 2 (CRLK1/2) on the plasma membrane (Yang et al. 2010; Zhao et al. 2017). CRLK1 and CRLK2 initiate a MEKK1–MKK1/2–MPK4 cascade to antagonize the MKK4/5–MPK3/6 pathway, leading to activation of ICE1 and expression of *CBF* genes (Li et al. 2017; Zhao et al. 2017). Accordingly, CBF proteins regulate the expression of *COR* genes and generate cryoprotectants, resulting in the acquisition of freezing tolerance. Activation of the MPK3/6 pathway is likely restricted to the cytosol during the early stages, whereas it promotes degradation of ICE1 in the nucleus at a later stage (Liu and Zhou 2018). These studies have proposed a model to account for how plants transmit a cold signal from the plasma membrane to the CBF-regulated *COR* genes during cold acclimation. Furthermore, MYB15 is a cold-inducible transcription factor and its transcriptional activity peaks after that of *CBFs*. MYB15 represses expression of the *CBFs* by directly binding to MYB recognition sites in *CBF1, CBF2*, and *CBF3* promoters (Agarwal et al. 2006). It is also possible that MAPK signaling regulates the MYB15 protein, suggesting a regulatory network upstream of CBFs during cold acclimation (Kim et al. 2017a). In addition, activation of cold-responsive protein kinase 1(CRPK1) occurs on the plasma membrane. CRPK1 phosphorylates 14-3-3 proteins that represent a family of highly conserved regulatory proteins in eukaryotes. In *A. thaliana*, phosphorylation of the κ and λ isoforms of 14-3-3 proteins promotes their shuttle from the cytosol to the nucleus, where they interact with and destabilize CBF proteins (Liu et al. 2017).

Calmodulin binding transcription activator (CAMTA) transcription factors are positive regulators of *CBFs* (Doherty et al. 2009). CAMTA1, CAMTA2, CAMTA3, and CAMTA5 induce expression of the *CBFs* within minutes in response to low temperature. CAMTA1, CAMTA2, CAMTA3, and CAMTA5 have been reported to directly bind to the *CBF2* promoter (Doherty et al. 2009; Kim et al. 2013; Kidokoro et al. 2017). However, the *camta3* mutations alone, as well as *camta1, camta2, camta4, camta5*, and *camta6* alone, do not show reduced freezing tolerance compared to wild type, indicating that cold acclimation requires the combined function of at least two members of the CAMTA family (Doherty et al. 2009; Kidokoro et al. 2017). Under natural conditions, low temperature can either occur as a sudden temperature drop (e.g., cold shock during the night or under abnormal weather conditions) or as a gradual temperature decrease (e.g., temperature change from autumn to winter). Although the expression of *CBF* and *COR* genes occurs during both rapid and gradual temperature decreases, different signaling pathways may be involved. Recent studies indicated that CAMTA3 and CAMTA5 regulate the expression of *CBF1* and *CBF2* during the day and night in response to a rapid but not slow temperature decrease, suggesting that CAMTA3 and CAMTA5 may function in cold shock signaling but not in the temperature change from autumn to winter (Kidokoro et al. 2017). The activation mechanisms of CAMTAs and how they interconnect with circadian regulation of *CBFs* during cold acclimation requires further study.

## CBF–*COR* pathway functions in other plants than *A. thaliana*

CBF genes have been identified in a range of plant species, ranging from grasses to trees (Puhakainen et al. 2004; Benedict et al. 2006; Tondelli et al. 2011). The initiation of cold acclimation in trees involves extensive reprogramming of gene expression that has been reported to include functional *CBF* genes (Puhakainen et al. 2004; Benedict et al. 2006; Welling and Palva 2008; Menon et al. 2015). For instance, in poplar, *PtCBF1, PtCBF2, PtCBF3*, and *PtCBF4* are induced at 5 °C in leaves, whereas only *PtCBF1* and *PtCBF3* show significant induction in stems. In leaves, *PtCBF1* and *PtCBF2* transcript levels peak 8 h after transfer to 5 °C, and *PtCBF3* and *PtCBF4* transcript levels peak at 3 h (Benedict et al. 2006). Overexpression of a *CBF* gene from *A. thaliana* in other plant species or overexpression of *CBFs* from other species in *A. thaliana* confers increased freezing tolerance. It also induces expression of CBF-regulated *COR* genes, indicating that the function of CBF genes is widely conserved in higher plants (Benedict et al. 2006; Tondelli et al. 2011). Furthermore, overexpression of a *CBF* gene, e.g., in apple, barley, potato, and poplar, enhances freezing tolerance even without cold acclimation. The enhanced freezing tolerance in transgenic plants is accompanied by the induction of *COR* genes (Benedict et al. 2006; Pino et al. 2008; Wisniewski et al. 2011; Jeknic et al. 2014; Soltesz et al. 2013; Park et al. 2015). In birch and poplar, freezing tolerance is reached after several weeks of cold acclimation in which *CBF* genes are affected by both photoperiod and day/night temperature cycling, indicating that *CBF* genes are functional in cold acclimation under natural conditions (Puhakainen et al. 2004; Welling and Palva 2008). However, the initiation of cold acclimation under natural conditions and responses by plants for the survive of seasonally low winter temperatures are complex and the key function of the CBF–*COR* pathway in these processes requires further study.

## Potential impact of climate change on signal transduction of cold acclimation

**Fig. 2:**
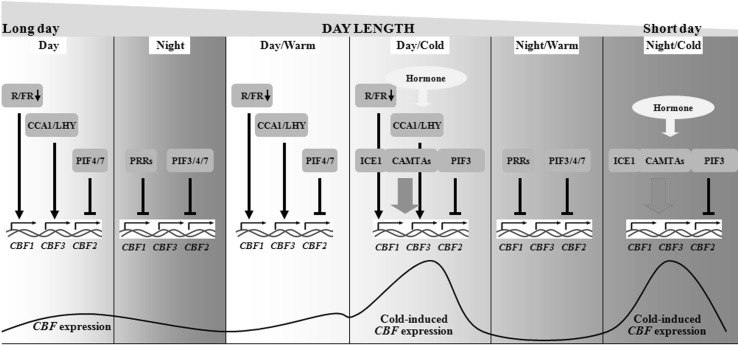
Expression of *CBFs* regulated by light quality, the circadian clock, and photoperiod. Under warm daytime, a decrease in the R/FR ratio leads to increased CBF expression under long-day or short-day conditions. CCA1 and LHY directly bind to CBF promoters to positively regulate CBF expression in the early morning. PhyB and the activity of PIF4/7 repress CBF expression by directly binding to the promoter region, whereas PIF3 is degraded by EBF1/2. During warm night, *CBF* expression is inhibited by PRRs and PIF3/4/7. Under short-day conditions, cold stress can occur during the day or night. ICE1 can be activated to induce *CBF* expression. CAMTA3 and CAMTA5 regulate the expression of *CBF1* and *CBF2* in response to a rapid temperature decrease. PIF3 represses CBF expression under cold conditions during day and night to balance CBF expression. The expression of CBF is also regulated by chloroplast signals and hormones. *CAMTAs* calmodulin-binding transcription activators, *CBF* C-repeat binding factor; CCA1, circadian clock-associated 1, *ICE1* inducer of CBF expression 1, *LHY* late elongated hypocoty l, *PIF* phytochrome-interacting factor, *PRRs* pseudo-response regulators, *R/FR* red to far-red ratio

**Fig. 3:**
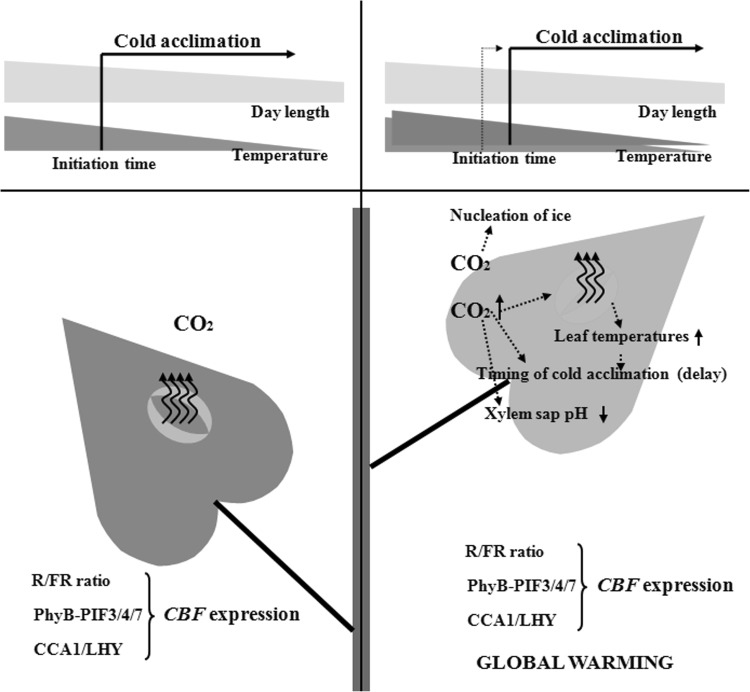
Schematic illustration of the impact of climate change on cold acclimation. Cold acclimation is caused by a complex interaction between a decreasing photoperiod and decreases in temperature. Climate change can delay the time of cold acclimation, and cold acclimation will be affected by erratic temperature events. Global warming can directly reduce the effectiveness of cold acclimation by disrupting the combined effects of photoperiod and temperature. Elevated CO_2_ concentration affects plant cold acclimation and freezing tolerance by nucleating ice in cells, increasing leaf temperatures, delaying the timing of cold acclimation, and changing xylem sap pH. The increase in leaf temperatures may affect membrane fluidity and the activity of calcium channels, and, thus, subsequent cellular signaling. Changes in xylem sap pH may affect the chemical characters of several *COR*-gene products and ABA signaling. Elevated CO_2_ concentration can affect both the timing and rate of cold acclimation in combination with warmer temperatures, shorter photoperiod, and lower irradiance. *CBF* C-repeat binding factor, *CCA1* circadian clock-associated 1, *CO*_*2*_ carbon dioxide, *LHY* late elongated hypocoty l, *PhyB* phytochrome B, *PIF3/4/7* phytochrome-interacting factor 3, 4 and 7, *R/FR* red to far-red ratio

Under natural autumn conditions, most temperate plants acquire cold acclimation by detecting the complex interaction between decreasing photoperiod and decreasing temperature (Rapacz et al. 2014; Maurya and Bhalerao 2017). Both timing and rate of cold acclimation are critical for freezing tolerance and successful overwintering. The expression of *CBF* genes is affected by both light quality and photoperiod (Fig. 2). With increasing temperature, cold acclimation will occur later in autumn or early winter with shorter photoperiods and lower total irradiance. Therefore, global warming can directly reduce the effectiveness of cold acclimation by disrupting the combined effects of photoperiod and temperature (Fig. 3). Indeed, at high latitudes, freezing tolerance of perennial grasses is impaired when cold acclimation occurs during warmer extended autumns (Dalmannsdottir et al. 2017). Another feature of temperature change in a changing climate is the frequency and severity of erratic temperature events. Disorganized cold acclimation causes higher susceptibility of plants to erratic temperature events. Erratic temperatures affect the plant freezing tolerance is mainly through deacclimation and reacclimation, two processes that also include expressions of *COR* genes and *CBF* genes (Kovi et al. 2016; Pagter and Arora 2013).

Cold acclimation is correlated to the ability to resist pathogens. At warmer temperatures (22 °C), CAMTA3 inhibits salicylic acid (SA)-mediated immunity in healthy plants. During cold acclimation, however, repression of the SA immunity by CAMTA3 can be overcome (Kim et al. 2013, 2017b). Therefore, CAMTA3-mediated cold acclimation not only contributes to subsequent freezing tolerance but also to SA-mediated immunity. Later in autumn, global warming disrupts cold acclimation and also cold acclimation-associated plant immunity. Since global warming favors survival of pathogens later in autumn (Newton et al. 2012), climate change can ultimately expand the opportunities for disease outbreak in particular plant species.

Elevated CO_2_ levels increase leaf temperatures mainly due to CO_2_-induced decrease in stomatal conductance during the day (Fig. 3; Ruiz-Vera et al. 2015). The increase in leaf temperature affects membrane fluidity and the activity of calcium channels that have been shown to activate CRLK1/2 and downstream MAPK signaling (Fig. 1). Elevated CO_2_ has been suggested to change xylem sap pH, which affects the chemical characteristics of several *COR*-gene products (Fig. 3). Changes in xylem sap pH have been suggested to increase ABA, which plays a role in the development of freeze tolerance (Eremina et al. 2016). Furthermore, the profound effect of elevated CO_2_ on cold acclimation originates from its combined effect with warmer temperature, shorter photoperiod, and lower irradiance (Fig. 3). Elevated temperature and CO_2_ during autumn and a shorter photoperiod have been reported to stimulate late-season net photosynthesis while impairing freezing tolerance in *Pinus strobus* seedlings (Chang et al. 2016).

Climate change affects the geographical plant distribution. The CBF pathway has been shown to be involved in local adaptation in *A. thaliana* during evolution. Analyses indicated that accessions collected from relatively warm environments express lower levels of *CBF* genes and downstream *COR* genes following cold acclimation compared to accessions from relatively lower winter temperature environments (Zhen and Ungerer 2008; Kang et al. 2013; Gehan et al. 2015). This difference occurs because southern accessions harbor more singletons in the promoter and coding regions of *CBF* genes. Long-term repression of the CBF pathway in climatic regions where plants experience low temperatures but not freezing stress might be advantageous and provide a driver for selection, as it has been shown that *CBFs* delay plant growth (Achard et al. 2008; Park et al. 2015). It seems that there is a trade-off of allocation of energy and nutrient resource allocation between plant growth and freezing tolerance (Hoermiller et al. 2017).

The CBF genes have been identified in numerous temperate plant taxa (Puhakainen et al. 2004; Benedict et al. 2006; Tondelli et al. 2011; Guo et al. 2018; Shi et al. 2018). In each particular plant species, at least one CBF gene can be induced in response to low temperature. However, despite conservation of CBF genes, plants do not show the same acquisition of freezing tolerance after cold acclimation. Overall, defective functioning of the CBF pathway could evolve through a mutation in the promoter (affecting *CBF* expression) or a mutation in a coding region (affecting binding of CBF to promoters of *COR* genes or affecting CBF stability). Therefore, cold responses in plants that do not acclimate to the cold are not strictly related to the expression of *CBF* genes. The CBF pathway has been reported to be involved in local adaptation in *Arabidopsis* during evolution. Analyses have indicated that accessions collected from relatively warm environments express lower levels of *CBF* genes and downstream *COR* genes following cold acclimation when compared to accessions from relatively lower winter temperature environments (Zhen and Ungerer 2008; Kang et al. 2013; Gehan et al. 2015). This difference may occur because the southern accessions harbor more singletons in the promoter and coding regions of the *CBF* genes. Long-term repression of the CBF pathway in climatic regions where plants might experience low temperatures but not freezing stress might be advantageous and provide a driver for selection, as it has been shown that *CBFs* retard plant growth (Achard et al. 2008; Park et al. 2015). Recent studies revealed a trade-off of allocation of energy and nutrient resources between plant growth and freezing tolerance (Hoermiller et al. 2017). Further studies are expected to investigate whether divergence in the *CBF* gene family among populations of other than *A. thaliana* plays an important role in the adaptive variation of cold acclimation in different geographic regions. Studies on the CBF pathway will have important implications for the expansion of plant ranges, invasiveness, and adaptation to novel climates.

## Conclusion and future perspectives

Cold is a major abiotic factor that affects plant growth, development, and survival on a daily and seasonal basis. The effects become more complicated due to the impact of climate change. Plants acquire freezing tolerance by cold acclimation, indicating that cold stress is perceived by plant cells. Recent studies reported the function of MAPK–ICE1 signaling in the regulation of CBFs (Fig. 1). Nevertheless, ICE1 is just one of the regulators of *CBF* genes. The elaborate mechanisms and possible regulatory networks upstream of the expression of *CBF* genes require further investigation. Furthermore, since the expression of *CBFs* is regulated by light quality, the circadian clock, and photoperiod (Fig. 2), understanding the daily and seasonal regulation of *CBFs* is necessary. In addition, *COR* gene expression is regulated by CBF-independent pathways and cold acclimation depends on *COR* gene-independent responses. Descriptions of the *COR* gene-independent responses and CBF-independent expression of *COR* genes are rare and further work is required.

Although CBF-dependent signaling has been demonstrated to be the major pathway to regulate the expression of *COR* genes, *COR* gene expression is also regulated by CBF-independent pathways. Furthermore, cold acclimation also depends on *COR* gene-independent responses (Fig. 1). Descriptions of the *COR* gene-independent responses and CBF-independent expression of *COR* genes are scarce and further responses should be documented. Moreover, it has been revealed that organelles and possibly the vacuole can also sense cold signals to modulate cellular metabolism and the proteome composition (Moellering et al. 2010). In addition, signaling transduction during cold acclimation is made even more complex by retrograde signals, whereby gene expression in the nucleus, chloroplast, and mitochondria must be coordinated depending on the status of the cell as a whole. Further studies are required to reveal the mechanisms of organelles and retrograde signaling during cold acclimation.

Due to increasing global temperatures in association with increasing atmospheric concentrations of CO_2_, future winters are expected to be milder. However, this change seems to harm plants as it disrupts cold acclimation and freezing tolerance (Fig. 3). Climate change can affect cold acclimation through the CBF–*COR* signaling pathway. Natural variation during cold acclimation has been shown to be associated with the geographical distribution of plants. Plants in climatic regions with low temperatures but not freezing stress tend to have evolved a defectively functioning CBF pathway, indicating that the CBF pathway is involved in local adaptation. More studies are required to measure the impact of climate change on cold acclimation at the molecular level. Although many questions remain unanswered, further research will expand our understanding of the signal transduction and regulation underlying cold acclimation in plants.

### Author contribution statement

YL and CH conceived and designed the review. YL, PD, and LL wrote the manuscript. All authors read and approved the manuscript.
